# Low affinity PEGylated hemoglobin from *Trematomus bernacchii*, a model for hemoglobin-based blood substitutes

**DOI:** 10.1186/1471-2091-12-66

**Published:** 2011-12-20

**Authors:** Daniela Coppola, Stefano Bruno, Luca Ronda, Cristiano Viappiani, Stefania Abbruzzetti, Guido di Prisco, Cinzia Verde, Andrea Mozzarelli

**Affiliations:** 1 Institute of Protein Biochemistry, CNR, Naples, Italy; 2 Department of Biochemistry and Molecular Biology, University of Parma, Parma, Italy; 3 Department of Physics, University of Parma, NEST Istituto Nanoscienze-CNR, Parma, Italy; 4 Department of Biotechnology, University of Verona, Verona, Italy

## Abstract

**Background:**

Conjugation of human and animal hemoglobins with polyethylene glycol has been widely explored as a means to develop blood substitutes, a novel pharmaceutical class to be used in surgery or emergency medicine. However, PEGylation of human hemoglobin led to products with significantly different oxygen binding properties with respect to the unmodified tetramer and high NO dioxygenase reactivity, known causes of toxicity. These recent findings call for the biotechnological development of stable, low-affinity PEGylated hemoglobins with low NO dioxygenase reactivity.

**Results:**

To investigate the effects of PEGylation on protein structure and function, we compared the PEGylation products of human hemoglobin and *Trematomus bernacchii *hemoglobin, a natural variant endowed with a remarkably low oxygen affinity and high tetramer stability. We show that extension arm facilitated PEGylation chemistry based on the reaction of *T. bernacchii *hemoglobin with 2-iminothiolane and maleimido-functionalyzed polyethylene glycol (MW 5000 Da) leads to a tetraPEGylated product, more homogeneous than the corresponding derivative of human hemoglobin. PEGylated *T. bernacchii *hemoglobin largely retains the low affinity of the unmodified tetramer, with a p50 50 times higher than PEGylated human hemoglobin. Moreover, it is still sensitive to protons and the allosteric effector ATP, indicating the retention of allosteric regulation. It is also 10-fold less reactive towards nitrogen monoxide than PEGylated human hemoglobin.

**Conclusions:**

These results indicate that PEGylated hemoglobins, provided that a suitable starting hemoglobin variant is chosen, can cover a wide range of oxygen-binding properties, potentially meeting the functional requirements of blood substitutes in terms of oxygen affinity, tetramer stability and NO dioxygenase reactivity.

## Background

Hemoglobin-based oxygen carriers (HBOCs) are a novel therapeutic class consisting of hemoglobin (Hb) derivatives administered intravenously as substitutes for blood transfusions. Modifications of the natural tetramer are required to reduce toxicity, as unmodified, cell-free Hb, once dissociated into dimers, is easily filtered by the kidneys and causes severe nephrotoxicity. Moreover, Hb extravasates through the endothelium, where it scavenges the vasoactive mediator nitrogen monoxide (NO), causing a range of toxic effects that include vasoconstriction and blood pressure increase. The strategies so far explored to avoid such effects mainly aim at increasing the molecular size of the natural Hb tetramers, thus limiting the size-dependent vessel extravasation and renal ultrafiltration. Beside some attempts at designing recombinant Hbs with higher molecular weight or lower dimer-tetramer dissociation constants [1-5], most products proposed for clinical use consist of Hb purified from whole blood and chemically modified to achieve either intramolecular cross-linking or conjugation with polyethylene glycol (PEG) [6]. PEG derivatization usually consists in the reaction between maleimido-functionalized PEG (MAL-PEG) molecules with either solvent-exposed cysteyl residues or thiol groups introduced through the reaction of lysyl side chains with 2-iminothiolane (IMT) under either aerobic [7] or anaerobic [8] conditions. PEG-decorated human Hb (HbA) derivatives have been evaluated in several clinical trials [9]. Adverse effects have so far prevented their application as a replacement of red blood cells [10].

One of the limits of HBOCs lies in the large differences between their oxygen binding properties with those of red blood cells. As a matter of fact, cell-free HbA cannot bind the intra-erythrocyte allosteric effector 2,3-bisphosphoglycerate, which increases the *P*_50 _(the oxygen partial pressure required to achieve half saturation) from 10 Torr to around 26 Torr at 37°C, pH 7.4. Moreover, free Hb in the plasma is usually at concentrations low enough to significantly dissociate into dimers, which do not show cooperativity and exhibit a *P*_50 _close to that of R-state Hb. PEGylation itself destabilizes the Hb tetramer and shifts the tetramer-dimer equilibrium towards the latter, with loss of cooperativity and a further increase in affinity [11]. Particularly, the reaction of PEG with Cys β93, conserved in 90% of vertebrates [12], was associated with tetramer dissociation and increased affinity [11,13]. As a matter of fact, both PEGylation of HbA in the T quaternary state, where Cys β93 is not reactive [8] and the reversible protection of Cys β93 HbA prior to conjugation [14] result in higher tetramer stability and lower affinity. However, based on experiments on HbA mutants, an increase in oxygen affinity seems to be at least partially independent of the derivatization of Cys β93 [15], suggesting that PEGylation induces changes in the hydration shell of hemoglobin, shifting the conformational equilibrium towards the more hydrated R state, regardless of the PEGylation sites.

In the light of the recent setbacks suffered by PEGylated Hb in clinical trials [10], a deeper investigation of the relationship between the oxygen-binding properties and PEGylation in Hbs was undertaken. One of the possible strategies focused on the use of non-human PEGylated Hbs, taking advantage of the low immunogenicity of PEGylated proteins in general [16]. Non-human Hbs might greatly differ in terms of PEGylation pattern, oxygen-binding properties and sensitivity to allosteric effectors. A product consisting of bovine Hb decorated with 10-12 units of 5000 Da-MW PEG was investigated as a possible blood substitute and showed a *P*_50 _of 10.2 Torr at 37°C [17], higher than that of PEGylated human Hb but still far from that of human blood (around 26 Torr). TetraPEGylated canine Hb [16] similarly showed a *P*_50 _of 10 Torr under the same conditions. In view of investigating the relationship between the oxygen affinity of animal Hbs and that of their PEGylation products, Hbs from Notothenioidei, the dominant suborder of teleosts in Antarctica, are particularly interesting, as they show peculiar features that make them potentially less sensitive to the undesirable effects of PEGylation. The oxygen affinity of these Hbs is exceptionally low [18], an evolutionary consequence of the high oxygen concentration in the cold Antarctic waters. Moreover, unlike HbA, fish Hbs show little or no dissociation of the tetramer into dimers, even in the ligated form [19]. Finally, Cys β93, present in the great majority of vertebrate Hbs and known to greatly perturb the properties of PEGylated Hbs and to scavenge NO [11], is missing in Hbs of almost all teleosts. The remaining cysteyl residues are all buried inside the protein matrix [20], suggesting that PEG conjugation can be carried out regardless of the quaternary or ligation state. We therefore decorated Hb from *Trematomus bernacchii (Tb*Hb*) *with PEG and characterized the reactivity with oxygen and NO. The results were compared with those obtained for PEGylated HbA.

## Methods

### Reagents

2-iminothiolane (IMT), HEPES buffer, ethylendiaminotetraacetic acid (EDTA), phosphate buffered saline solution (PBS), sodium ascorbate, catalase and the reagents for the Hayashi enzymatic reducing system [21] were purchased from Sigma Chemical Co. (St. Louis, MO, U.S.A.) and maleimido polyethylene glycol (MAL-PEG) (5600 Da-MW) from Nektar Molecule Engineering (Nektar Therapeutics, San Carlos, CA, U.S.A.). All other reagents were of the best available commercial quality.

### Collection of specimens and Hbs purification

Specimens of *Tb*Hb were collected by gill nets or hook-and-line in the vicinity of Terra Nova Bay "Mario Zucchelli" Station (74°42'S, 164°07'E), Ross Sea, Antarctica, and kept in aquaria supplied with running, aerated sea water. Blood was withdrawn with heparinised syringes from the caudal vein. Hemolysates were prepared as described previously [22]. Saline-washed erythrocytes were frozen at -80°C until use. Purification of *Tb*Hb at 98% was carried out as described previously [23]. HbA was purified as described elsewhere [24].

### Cysteine titration

Preliminarily to PEGylation experiments, the reactivity of the cysteyl residues of *Tb*Hb was evaluated in both the deoxy- (T) and carboxy- (R) states using 4,4'dithiodipyridine (4-PDS) [25]. For the titration under anerobic conditions, the protein solution was incubated under helium flux until the absorption spectrum shifted to the deoxy-Hb form. A separately deoxygenated stock solution of 4-PDS was anaerobically added.

### Hbs PEGylation

**Figure 1:**
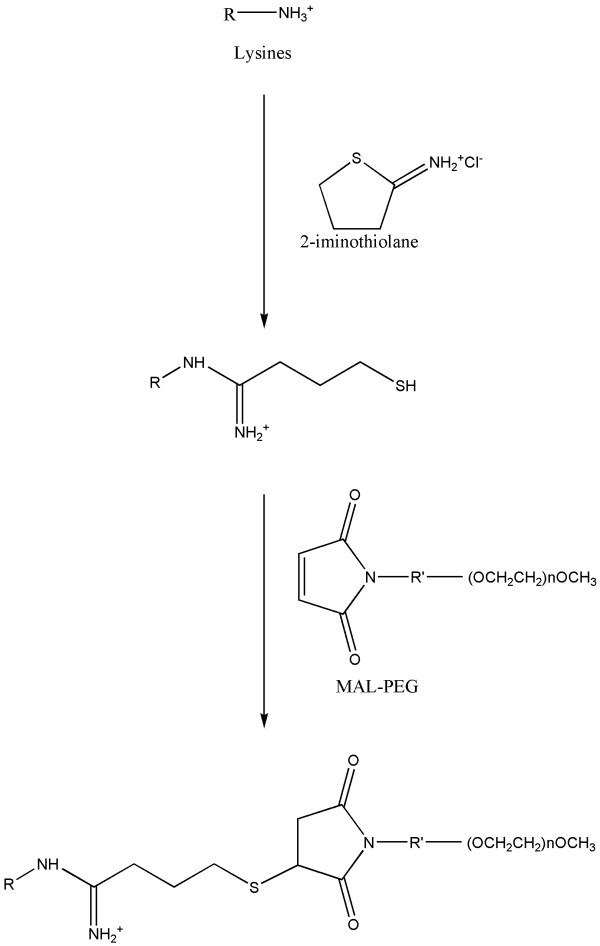
**Schematic representation of extention arm facilitated PEGylation chemistry**.

The PEGylation reaction was carried out following the protocol published for HbA in aerobic conditions [11,26]. Briefly, *Tb*Hb or HbA were treated in the presence of IMT (80 moles/tetramer moles) and then with MAL-PEG 5600 Da-MW (12 moles/tetramer moles) (Figure 1), at 10°C to prevent any heme oxidation during the reaction. *Tb*Hb was treated in the presence of CO, subsequently removed under oxygen flow before measurements. The reactions of IMT and MAL-PEG were quenched using lysine and cysteine in excess, respectively. Less than 5% met-Hb was formed during the reaction. To monitor the PEGylation reaction, small aliquots of the reaction mixture were sampled every 10 minutes. The reaction was quenched by addition of lysine and cysteine in excess. The samples were analyzed by sodium dodecylsulfate/polyacrylamide gel electrophoresis (SDS-PAGE) and the electropherograms were evaluated using the Quantity One software (Bio-Rad). Under denaturing conditions, SDS-PAGE applied to PEGylated Hbs was able to separate PEGylated Hb into unmodified globin chains and globin chains with different PEGylation degree [11]. To evaluate the homogeneity of the final products, electrophoresis under native conditions was carried out in 8-2% gradient gel and analyzed as described elsewhere [27].

### Determination of oxygen-binding curves

Oxygen-binding curves of HbA, *Tb*Hb and their PEGylated derivatives, called PEG-Hb^oxy ^and PEG*Tb*Hb, respectively, were measured with a modified tonometer [24]. Before titration, the stock solutions of the proteins were diluted in a solution containing 100 mM HEPES, 1 mM sodium EDTA, at either pH 7.0 or 8.0, to a final concentration of 30 μM. Sodium ascorbate and catalase were added to final concentrations of 5 mM and 10^3 ^U/ml, respectively, to prevent significant autoxidation during the measurement. For *Tb*Hb and PEG*Tb*Hb, experiments were carried out in the presence and absence of the allosteric effector ATP at a final concentration of 3 mM. For the experiments on *Tb*Hb and PEG*Tb*Hb stored in the carbomonoxy form, CO was removed by exposure to pure oxygen for 2 hours prior to titration, taking advantage of the relatively low affinity of *Tb*Hb for CO (data not shown). The samples where then exposed to oxygen partial pressures ranging from 0 to 760 Torr generated using an Environics 4000 (Environics inc, Tolland, CT, U.S.A.) gas mixer and pre-mixed helium/oxygen bottles, at 10°C. Spectra were collected in the 350-700 nm range using a Cary 4000 (Agilent Technologies, Lexington, MA, U.S.A.) spectrophotometer. The oxygen saturation at each partial oxygen pressure was determined by deconvoluting the spectra in the 450-700 nm range to a linear combination of the reference spectra of deoxy-, oxy- and met-Hb, plus a baseline. The deoxy reference spectra were obtained for HbA and *Tb*Hb in the presence of sodium dithionite, whereas reference spectra for the oxy forms were obtained in pure oxygen in the presence of the Hayashi reducing system [21]. The Hill's coefficient (n) and *P*_50 _were calculated by linear regression of the Hill's plots in the saturation range 20-80%.

### Flash photolysis experiments

The experimental set up has been described previously [28,29]. Flash photolysis measurements were performed using the circularly polarized second harmonic of a Q-switched Nd:YAG laser (Surelite II Continuum) and a cw Xe arc lamp as a monitoring beam. The transient absorbance signals were measured at 436 nm with a 5-stage photomultiplier.

### NO dioxygenase activity

The rates of the NO dioxygenase reactivity at a single NO concentration were determined for HbA, *Tb*Hb, PEG-Hb^oxy ^and PEG*Tb*Hb by rapid mixing using a stopped-flow apparatus (SX.18MV, Applied Photophysics). The NO solutions were generated by equilibrating a previously deoxygenated PBS solution at pH 7.4 with a gas mixture of NO in nitrogen. The exact concentration of NO was then measured by titration of the solution with deoxygenated HbA under anaerobic conditions and determined to be 12 μM. The protein concentration was 3 μM. The reaction was monitored at 405 nm. Between 5 and 10 traces were collected and averaged. All measurements were carried out under strict anaerobic conditions at 20°C.

## Results and Discussion

### Cysteine reactivity

Sulfhydryl reactivity towards 4-PDS of carbomonoxy- and deoxy- *Tb*Hb was very slow (data not shown) with the fastest-reacting cysteine completing the reaction in more than 24 hours. The slow reactivity of *Tb*Hb confirmed the structural data [20], which indicated the absence of exposed cysteyl residues. 2-iminothiolane-generated SH groups are therefore predicted to be the only reactive sites towards MAL-PEG (Figure 1). For comparison, HbA reacts with a twice equimolar amount 4-PDS within 10 minutes (data not shown) due to the exposed Cys β93. It is therefore expected that *Tb*Hb, unlike HbA, would not react directly with MAL-PEG.

### PEGylation

**Figure 2:**
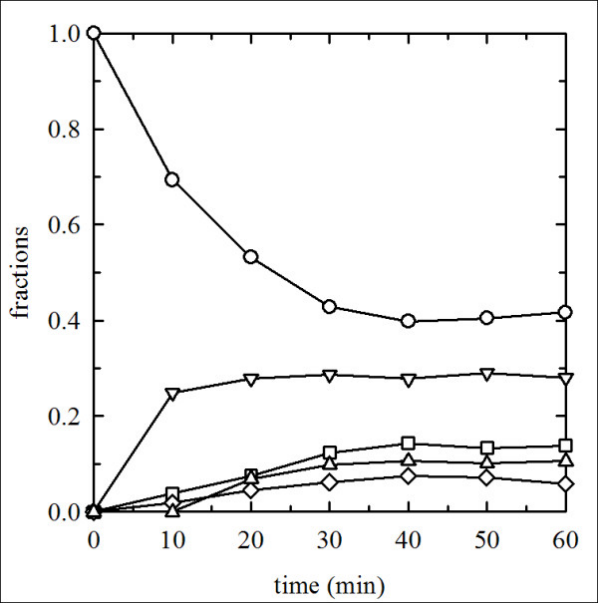
**PEGylation reaction rates**. Reaction time dependence of PEG conjugation on globin subunits as determined from the densitometric analysis of SDS-PAGE gels: 0 PEG bound per subunit (open circles), one PEG bound per subunit (open inverted triangles), two PEG bound per subunit (open squares), three PEG bound per subunit (open diamonds), four PEG bound per subunit (open triangles).

**Figure 3:**
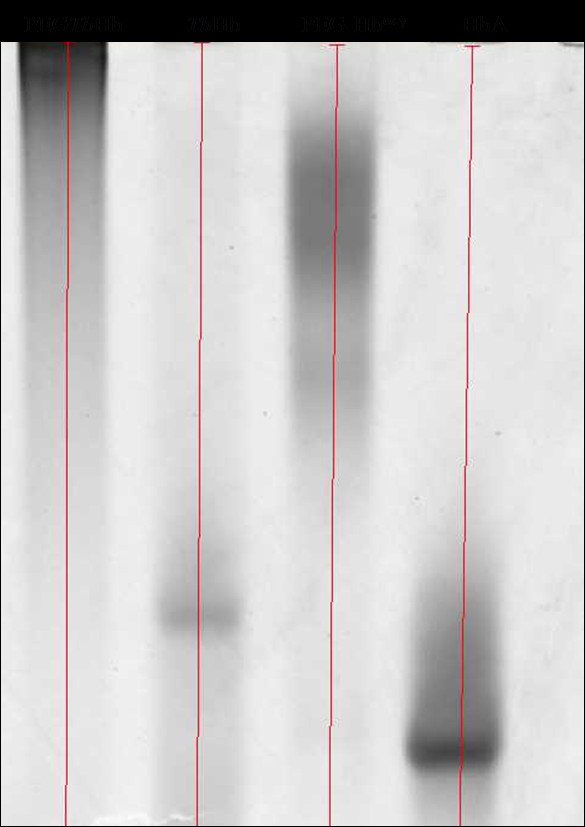
**Size distribution of derivatized hemoglobins**. Gradient native PAGE of HbA, *Tb*Hb and their PEGylated derivatives.

Samples collected at different times of the PEGylation reaction were compared in a SDS-PAGE gel (data not shown) and analyzed by densitometry (Figure 2). The reaction appeared to be completed in 30 minutes. The densitometric analysis [11] showed that about four PEG chains per tetramer are added during the reaction, as compared to the 5-6 PEG chains/tetramer added to HbA under the same reaction conditions. Nevertheless, in native electrophoresis, PEG*Tb*Hb exhibited a slower migration with respect to PEG-Hb^oxy^, possibly due to differences in electric charge (Figure 3). Some other noticeable differences emerged with respect to PEG-Hb^oxy^. Particularly, the *Tb*Hb PEGylated derivative appeared more homogeneous and did not show any traces of unmodified tetramer (Figure 3), which were consistently observed in all preparation of PEG-Hb^oxy ^[27]. It is widely recognized that unmodified Hb is very toxic, as it can extravasate and be filtered at glomerular level. The complete derivatization of *Tb*Hb would therefore be a valuable property for a blood substitute.

### Oxygen-binding properties

**Figure 4:**
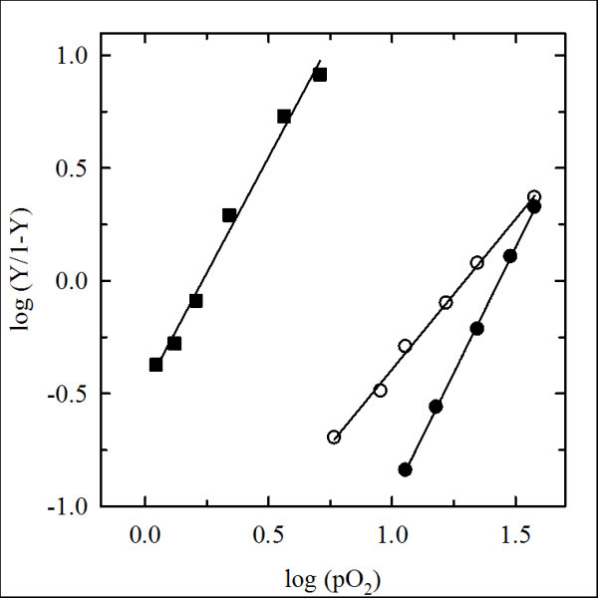
**Oxygen binding properties of derivatized hemoglobins**. Hill plots of oxygen-binding curves of HbA (closed squares), *Tb*Hb (closed circles) and PEG*Tb*Hb (open circles), measured in 100 mM HEPES 1 mM EDTA, 5 mM sodium ascorbate, 10^3 ^U/ml catalase, pH 7.0, at 10°C. Experimental points are fitted to the Hill equation, with calculated Hill's coefficients and *P*_50_'s reported in Table 1.

Oxygen affinity, cooperativity and the Bohr effect of PEGylated Hb derivatives and unmodified Hbs were measured under different conditions (Figure 4). The oxygen-binding curves were determined at pH 7.0 and pH 8.0, at 10°C. The analysis allows calculating *P*_50 _and Hill coefficient values (Table 1). At pH 7.0, HbA exhibited a *P*_50 _of 1.7 Torr, which decreased to approximately 0.4 Torr upon PEGylation (Table 1). The derivatization also resulted in loss of cooperativity, with the Hill coefficient decreasing from around 2 to 1.2. *Tb*Hb, under the same conditions, showed a much higher *P*_50 _of 28.2 ± 0.2 Torr. PEGylation resulted in an increase in oxygen affinity to 19.7 ± 0.3 Torr. However, *P*_50 _remained 50-fold higher than PEG-Hb^oxy ^under the same conditions. Cooperativity was significantly reduced, with the Hill coefficient decreasing from 2 to around 1.3. The changes in oxygen-binding properties of *Tb*Hb upon PEGylation are therefore similar to those observed for PEG-Hb^oxy^, in particular showing loss in cooperativity. Considering the stability of the *Tb*Hb tetramer, this effect is likely due to the steric effects of the PEG moieties, which prevent the transition between the T and R states, rather than the dissociation of the tetramer, as seen in HbA. Despite the loss in cooperativity, the *P*_50 _of PEG*Tb*Hb remains remarkably high. Moreover, ATP at saturating concentrations of 5 mM (data not shown) still acts as an allosteric effector (Table 1), raising *P*_50 _to 31.1 Torr. These data, combined with those relative to PEGylated Hbs of other species, particularly in bovine [30] and canine Hbs [16], show that, regardless of their different oxygen affinity, there is indeed a correlation between the increase in affinity and the PEGylation reaction. The non-specific effect of PEGylation in increasing the oxygen affinity was also demonstrated within the same preparation, through the electrophoretic separation of differently PEGylated HbA derivatives having different affinities [27].

**Table 1:** Oxygen binding parameters of human and T. bernacchii Hbs and their PEGylated derivatives

	pH 7.0		pH 8.0	
**Protein**	***P***_**50**_	**n**	***P***_**50**_	**n**

HbA	1.7 ± 0.1	2.05 ± 0.02	0.39 ± 0.3	1.72 ± 0.03

PEG-Hb^oxy^	0.4 [35]	1.2 [36]		

*Tb*Hb	28.2 ± 0.2	2.06 ± 0.01	7.3 ± 0.1	1.75 ± 0.03

PEG*Tb*Hb	19.7 ± 0.3	1.33 ± 0.02	5.3 ± 0.1	1.09 ± 0.02

PEG*Tb*Hb + ATP	31.1 ± 0.2	1.09 ± 0.01	4.4 ± 0.3	1.02 ± 0.03

Measurements were made in 100 mM HEPES, 1 mM EDTA, 5 mM ascorbate, 10^3 ^U/ml catalase, at 10°C. The values for PEG-Hb^oxy ^were calculated from published data taking into account the temperature dependence of the oxygen binding parameters.

### Flash photolysis experiments

**Figure 5:**
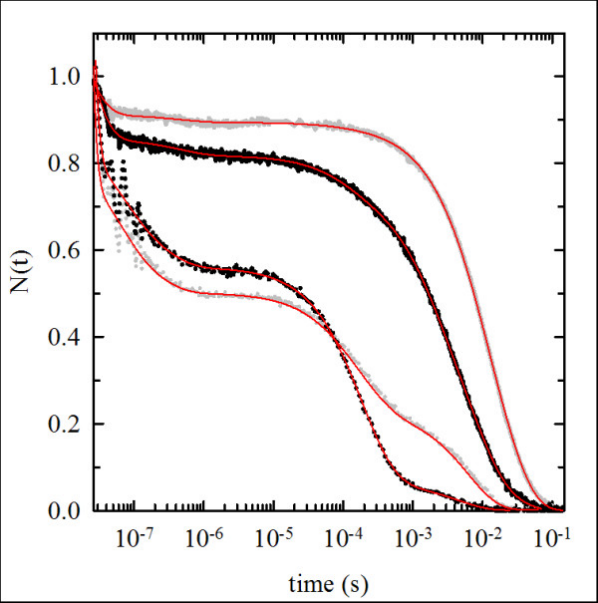
**CO rebinding properties of derivatized hemoglobins**. Effect of PEGylation on CO rebinding to HbA and *Tb*Hb and their PEGylated derivatives. The time courses of the deoxyheme fraction are shown for HbA (gray circles), PEG-Hb^oxy ^(black circles), *Tb*Hb (gray solid line) and PEG*Tb*Hb (black solid line), in 100 mM HEPES, 1 mM sodium EDTA, 1 atm CO, pH 7.0 at 10°C. Data were fitted as reported in Materials and Methods. The fitting curves are shown in red.

The CO rebinding kinetics measured after laser flash photolysis on the carbomonoxy forms of HbA and *Tb*Hb in the absence and presence of PEGylation is reported in Figure 5. The rebinding traces were converted to fraction N(t) of deoxy-Hb as a function of time. Dependence of the kinetics on the CO concentration allows distinguishing between unimolecular and bimolecular processes (data not shown). As it is well established for HbA, CO rebinding comprises multiple phases: a nanosecond (unimolecular) geminate process due to rebinding from within the heme pocket or the protein matrix, and two second order processes, one in the microsecond time scale, ascribed to bimolecular rebinding to quaternary R state, and one in the millisecond time scale, ascribed to the bimolecular rebinding to proteins that have switched to quaternary T state [31]. In order to reproduce the experimental rebinding curves for HbA, we used a sum of six exponential decays functions, as proposed by Kliger and coworkers [32]. In Figure 5 we also show the results of the global fitting on HbA and *Tb*Hb (both with and without PEGylation), demonstrating a very good agreement between calculated and experimental curves. Besides the two exponential decays which are necessary for describing the geminate phase (10 and 182 ns for HbA; 14 and 452 ns for *Tb*Hb), we detected two processes ascribed to quaternary relaxation from the R to the T states in the micro-millisecond time scale (1 and 140 μs for HbA; 110 μs and 1.5 ms for *Tb*Hb), one phase associated with the rebinding to R state (350 μs for HbA; 7 ms for *Tb*Hb) and one phase associated with the rebinding to T state (7 ms for HbA; 21 ms for *Tb*Hb). As previously shown in HbA [11], while PEGylation preserves the general features of the dynamics and reactivity of the protein, it partially prevents the R to T relaxation, so that upon PEGylation the fractional amplitude of the rebinding to R changes from about 27% to 80% in HbA and from 34% to 76% in *Tb*Hb.

### NO dioxygenase activity

**Figure 6:**
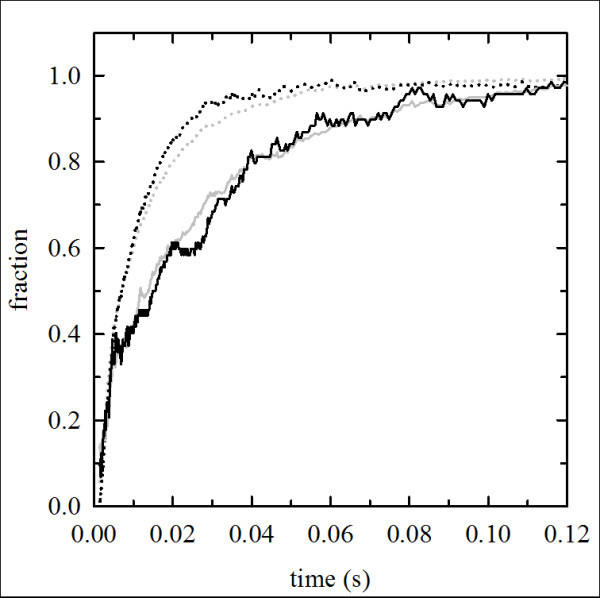
**NO dioxygenase reactivity**. Determination of the effect of PEGylation on NO dioxygenase reactivity of HbA and *Tb*Hb and their PEGylated derivatives. The reaction was carried out by rapid mixing under anaerobic conditions at 20°C. 3 μM oxygenated HbA (gray dotted line), *Tb*Hb (gray solid line), PEG-Hb^oxy ^(black dotted line), and PEG*Tb*Hb (black solid line), with a deoxygenated PBS solution containing 12 μM NO. The reaction was monitored at 405 nm and absorbance differences were normalized.

The scavenging of NO, associated with an extremely rapid dioxygenation reaction with oxy-Hb to form met-Hb and inert nitrate, is significant when Hb is present in blood vessels outside erythrocytes and is likely to be the main determinant of the adverse effects of HBOCs [33,34]. In order to develop a possible blood substitute, it is crucial to consider not only oxygen-transport properties of these Hbs, but also the influence that these products can have on NO homeostasis. The reaction rates of the NO with unmodified *Tb*Hb and its PEGylated Hb derivatives were measured and compared with those of HbA and its PEGylation product. Analysis of the oxidation kinetics of oxy *Tb*Hb treated with NO (Figure 6) yields a k_obs _of 7.2 μM^-1^s^-1^, indicating slower reactivity with respect to HbA, with a k_obs _of 64 μM^-1^s^-1 ^under the same experimental conditions, in agreement with literature data [34]. PEGylation of *Tb*Hb seems to only marginally affect the NO dioxygenase reactivity, with a k_obs _for PEG*Tb*Hb of 7.7 μM^-1^s^-1^. A comparable decrease in the NO dioxygenase activity of HbA was achieved by mutating residues αE11, βE11, and βB10 (2-15 μM^-1^s^-1^) [33]. These mutants showed reduced *in vivo *vasoactivity, directly correlated with the *in vitro *NO oxygenation rate. The same pattern is observed in HbA, the PEGylation of which, under aerobic and anaerobic conditions, yields k_obs _of 83 μM^-1^s^-1 ^(Figure 6) and 86 μM^-1^s^-1 ^(data not shown), respectively.

## Conclusions

The functional characterization of the PEG-conjugated derivative of the highly stable Hb tetramer of *T. bernacchii *confirms the non-specific effects of PEGylation already observed in human, bovine and canine Hbs, including an increase in oxygen affinity, a decrease in cooperativity and a reduction of the R- to T-quaternary switching upon flash photolysis. However, these non-specific effects are accompanied by the partial retention of the remarkably low affinity for oxygen, the sensitivity to allosteric effectors and the low NO dioxygenase reactivity. These results indicate that PEGylated Hbs, provided that a suitable starting Hb variant is chosen, can cover a wide range of oxygen-binding properties, potentially meeting the functional requirements of blood substitutes.

## Abbreviations

4-PDS: 4,4'dithiodipyridine; ATP: adenosine triphosphate; EDTA: ethylenediaminetetraacetic acid; HbA: human hemoglobin; HBOC: hemoglobin-based oxygen carrier; IMT: 2-iminothiolane; MAL-PEG: maleimido polyethylene glycol; PEG: polyethylene glycol; PBS: phosphate buffered saline; SDS-PAGE: dodecylsulfate/polyacrylamide gel electrophoresis; *Tb*Hb: *Trematomus bernacchii *hemoglobin.

## Authors' contributions

GdP initiated this investigation by collecting material from cold-adapted fish in Antarctica. DC, CiV and GdP purified *T. bernacchii *Hb. DC and LR performed Hb PEGylation and electrophoretic characterization, DC and SB investigated oxygen binding and performed cysteyl titrations. SB characterized NO dioxygenase reactivity and wrote the manuscript, CrV and SA performed the flash photolysis experiments. AM contributed to the manuscript preparation. All authors read and approved the final manuscript.
